# A novel prescription pedometer-assisted walking intervention and weight management for Chinese occupational population

**DOI:** 10.1371/journal.pone.0190848

**Published:** 2018-01-11

**Authors:** Yingxiang Yu, Yiran Lv, Bin Yao, Liguang Duan, Xiaoyuan Zhang, Lan Xie, Cuiqing Chang

**Affiliations:** 1 Institute of Sports Medicine, Peking University Third Hospital, Beijing, China; 2 Descom Information Technology (Beijing) Co. Ltd., Beijing, China; University of Alabama at Birmingham, UNITED STATES

## Abstract

**Background and aim:**

Information technology has been previously used for the research and practice of health promotion. Appropriate and effective health promotion methods used by professional groups remain to be investigated. This study aimed to assess the feasibility and effectiveness of a weight management program among the Chinese occupational population using and a novel information technology exercise prescription.

**Study design and participants:**

A 3-month open, self-monitored intervention trial, involving individualized pedometer-assisted exercise prescription and a one-time targeted dietary guidance prior to exercise was conducted on the Chinese occupational population aged 18–65 years in China from 2015 to 2016. Data were collected from March 2015 to May 2016 and analyzed from June 2016 to August 2016. Participants were also asked to synchronize exercise data of the pedometer to the Internet-based Health System Center daily (at least weekly), by connecting to the personal computer (PC) using a USB cable or via Bluetooth.

**Results:**

Eligible participants included 802 Chinese occupational persons, and 718 of them followed exercise interventions with 89.5% (718/802) adherence to the exercise programs. Of them, 688 participants completed the program with 85.8% (688/802) adherence to the exercise program and their data were analyzed. Weight decreased by 2.2% among all overweight/obese participants, with 1.8% reduction in waist circumference and 3.3% reduction in body fat percentage (p< 0.001). Weight and body fat percentage in normal-weight individuals decreased by 0.7% and 2.5%, respectively (p < 0.01). A weight gain of 1.0% was observed in all underweight participants (p< 0.05), and 68.2% (208/305) of overweight/obese participants experienced weight loss, with an average reduction of 3.5%, with 20.2% (42/208) of them achieving weight loss ≥5%. Blood pressure and fasting serum glucose decreased significantly in both the overweight/obese and the normal-weight individuals (p < 0.05). The incidence of hypertension was significantly lower and lifestyle behavior significantly improved (p < 0.05).

**Conclusion:**

The prescription pedometer-assisted walking intervention can effectively improve exercise adherence and manage weight. This approach was also effective in controlling the risk factors of weight-related chronic diseases.

**Trial registration:**

Chinese Clinical Trial Registry (ChiCTR) ChiCTR-OOh-16010229

## Introduction

Weight is an important indicator of health. Being underweight or overweight is associated with an increased risk of adverse health outcomes [1]. In the past decades, in addition to being underweight, the development of diseases associated with being overweight has become a common problem globally. A pooled-analysis of ≥ 19.2 million participants in 186 countries from 1975 to 2014 demonstrated a rapid increase in the prevalence of overweight and obesity in both developed and developing countries; in addition, the global prevalence of obesity (BMI ≥ 30kg/m^2^) firstly surpassed that of underweight (BMI < 18.5kg/m^2^) in 2004 in women and in 2011 in men [2]. A 2015 report on Chinese nutrition and chronic disease revealed a 30.1% and 11.9% prevalence of overweight and obesity among Chinese adults, respectively, representing a population of ≥ 55 million with overweight or obesity in China [3]. This problem is further aggravated by the fact that the Chinese population is rapidly ageing. With the accelerated aging of the population, the number of residents in China ≥ 60 years of age has increased each year, and the policy of delaying the retirement age is expected to be executed. In addition, the number of older employees is increasing. A major health problem for the occupational population is overweight/obesity and associated chronic diseases, such as hypertension, hyperlipemia, diabetes, and fatty liver, partly attributed to increased stress at work, sedentary lifestyle, and irregular diet with frequent eating out[4,5].

Several population-based studies have confirmed that moderate exercise and a balanced diet could help control weight and improve body composition [6,7,8,9]. However, effective delivery of health behavior interventions, particularly within the occupational population, remains to be explored. In this study, we evaluated the feasibility and effectiveness of an individualized prescription pedometer-based exercise and diet, supported by a well-established Internet-based Health System Center (IHSC), on weight control among employees. Furthermore, we aimed to provide evidence of the effectiveness of the IHSC-supported, prescription pedometer-based walking in remote weight management and prevention of the risk of chronic diseases in countries with a large population like China.

## Materials and methods

### Subjects and eligibility criteria

A total of 802 employees from institutions or enterprises from four areas in China (Beijing, Guangdong, Henan and Shaanxi) were recruited via advertisements at local health centers, hospitals, and commercial buildings. The study participants included 360 overweight and obese individuals, 398 normal-weight individuals and 44 underweight individuals.

The inclusion criteria were as follows: 1) full-time employees willing to manage their weight and improve their lifestyle and 2) aged 18–65 years. The exclusion criteria including the follows:1) secondary obesity; 2) history of weight-loss interventions (bariatric surgery, acupuncture, moxibustion, or prescribed medication), and significant changes (> 5%) in body weight in the past 3 months; 3) diagnosed osteoarthritis, abnormal physical deformities and history of spine or limb surgery or fracture in the past three months; 4) severe functional disorder or organic diseases of the heart, liver or kidney; 5) uncontrolled hypertension (blood pressure ≥ 180/110mmHg) or its complications; 6) fasting blood glucose ≥ 16.7mmol/L or diabetic complications; and 7) pregnant or lactating women.

### Study design and outcomes

This was a 3-month, self-monitoring, population-based study for weight management and chronic disease risk prevention. The primary outcomes were changes in body weight or body mass index (BMI), waist circumference (WC), and blood pressure (BP). Secondary outcomes included the changes in lifestyle behavior (scores), body fat percentage (BF %), fasting blood glucose (FBG), and serum lipid.

All participants underwent outcome measurements at baseline and at the end of the study period. All measurements were performed by well-trained professionals who completed the research program in accordance with standard operating procedures. Venous blood collection was performed by skilled and qualified nurses. All tests were performed at the study centers. Body weight and BF% were measured using BIA (Tanita MC-180, Japan) and accurate to 0.1 kg and 0.1%, respectively. BMI was calculated by dividing the weight by the square of height (kg/m^2^). WC was measured in accordance with the guidelines for prevention and control of overweight and obesity in Chinese adults [10], nearest to 0.1 cm. BP was measured with the right arm using a mercury sphygmomanometer, according to the procedures of 2010 Chinese Guidelines for the Management of Hypertension [11]. Laboratory data, including FSG, triglyceride(TG), total cholesterol (TC), low density lipoprotein-cholesterol(LDL-C), and high-density lipoprotein-cholesterol (HDL-C) were assessed using the automatic blood biochemical Analyzer (BACKMAN COULTER 5800, America). All instruments used in this study were calibrated before testing.

All participants were asked to complete a self-reported questionnaire, which was adapted from Healthstyle: A Self-Test, developed by the US Public Health Service. Assessment of health habits and lifestyle was interpreted using lifestyle behavior scores which were calculated by weighting six aspects of the behavior score: exercise, diet, smoking, drinking, stress handling and disease prevention. Each aspect was divided into four categories (out of 10 points): Excellent, 9–10; Good, 6–8; Fair, 3–5; and Poor, 0–2.

Data were collected from March 2015 to May 2016 and analyzed from June 2016 to August 2016. The protocol was approved by the Medical Ethics Committee of Peking University Third Hospital [NO.2014 (189–2)]. All enrolled participants signed informed consent forms. This study is registered and approved by the Chinese Clinical Trials Registry (ChiCTR registry number: ChiCTR-OOh-16010229) (http://www.chictr.org.cn/) after this trial had started a few months later. We believed that the time of clinical study registration did not affect any aspect of this study. First, this trial was authorized by the Medical Ethics Committee before enrolment of the participants. The intervention did not involve any drugs, and it did not have any adverse effects on the health of the participants. Futhermore, the intensity and duration of exercise are based on the global recommendations of physical activity for health by the World Health Organization and the Physical Activity Guideline for Chinese Adults.

### Intervention design

#### Health assessment

All participants underwent physical examination and blood biochemistry testing. These data, including information on medical history and treatment and lifestyle were recorded in the IHSC. The IHSC automatically generated a personalized exercise prescription and targeted dietary guidance in the form of web version for each participant.

#### Exercise intervention

Exercise prescription was mainly based on the global recommendations on physical activity for health of the World Health Organization[12], ACSM’s Guidelines for Exercise Testing and Prescription eighth edition[13] and the Physical Activity Guideline for Chinese Adults[14], which focused on vigorous walking, was supplemented by resistance training, stretching exercise and balance exercise programs. Personalized exercise and dietary prescriptions were provided to participants according to gender, age, BMI, physical activity level, metabolic status, medical history and other health information assessment. In addition, physical activity was divided into four levels, PAL0, PAL1, PAL2, and PAL3, according to different exercise habits at baseline. The exercise prescription included seven elements: type of exercise, exercise intensity (pace, steps/min), duration (≥ 10 min/session), frequency (5–7 days/week), daily exercise period (6:00–23:00), optimal timing (e.g., exercising 1.5–2 hours after a meal for participants with hyperglycemia or diabetes), and exercise tips of targeted groups. The intensity was equal to 3.0–6.0 metabolic equivalent (METs), and overall exercise time was 30-60min per day divided into three sessions, so that participants had the flexibility to complete all three sessions at once or in two or three parts. Prescription pedometers assist the participants to complete their exercise as required by the trial protocol.

The exercise prescription was downloaded to the pedometer, and was performed by participants and as a self-monitoring tool. The pedometer is intended to supervise and guide participants to strictly follow the requirements of the exercise prescription, in terms of intensity and duration. For example, if the pace was not satisfactory (less or higher than the prescribed pace), the walking time was <10 minutes, or the time of cessation >30 seconds during each exercise session, the pedometer would indicate that exercise task was not completed and needed to be repeated. The intervention period involed three courses of the exercise program. Each course lasted for 30 days. If the completion rate is ≥80% at the end of course, the following session is upgraded in terms of intensity and/or-) duration; if not, the same prescription setting will continue.

The participants were asked to regularly (daily or at least weekly) synchronize exersice data of the pedometer to IHSC, by connecting it to the PC via a USB cable or through the mobile via Bluetooth. Futhermore, investigators would check the data weekly and inform the participants if they failed to upload data on time or did not complete the exercise tasks by phone calls or text massages. We used IHSC, PC, APP, and prescription pedometer to continuously, dynamically, and remotely monitor the completion of the exercise.

#### Dietary guidance

The dietary recommendations generated by IHSC were based on the Chinese Dietary Reference Intakes (2013); [15], the Chinese Dietary Guidelines (2007);[16] and WHO Global Strategy on Diet, Physical Activity and Health[17]. It mainly consisted of equal-energy balanced diet or controlled- or low-energy balanced diet. Based on these diets, researchers assessed the main problems recorded in the dietary questionnaire on lifestyle or results of blood test to provided one-time targeted diet guidance before the exercise intervention. These dietary recommendations were in accordance with the 2010 Chinese Guidelines for the Management of Hypertension[11], 2013 China Guideline for Type 2 Diabetes, and Chinese guidelines on prevention[18] and treatment of dyslipidemia in adults[19].

#### Safety assessment

Adverse events mainly included injury due to improper exercise. However, any event occuring during the study period, that may result in failure to participate in the trial should be carefully recorded and promptly reported. The investigators must be notified of any adverse events related to the study intervention or implementation of the intervention, by phone or SMS within 24 hours of the adverse event. Since this study involved remote monitoring of the management, if participants did not report adverse events to the researchers, it was considered that there were no adverse events. Meanwhile, the participants who completed the exercise intervention were asked about the adverse events by investigators in person, and those who withdrew from the study were contacted by investigators to inquire about any adverse events. If no contact could be established, no adverse events were considered.

### Diagnostic criteria of overweight, obesity, hypertension, hyperglycemia and hyperlipidemia

We used BMI to assess obesity (BMI ≥28kg/m^2^), overweight (24.0–27.9kg/m^2^), and underweight (<18.5 kg/m^2^) [10]. Hypertension was defined as systolic blood pressure (SBP) ≥140 mmHg and/or diastolic blood pressure (DBP) ≥90 mmHg [11]; hyperglycemia was defined as FBG ≥6.1 mmol/L [18], and high serum lipid as TC ≥5.18 mmol/L(200 mg/dl), TG ≥1.76 mmol/L(150 mg/dl)or LDL-C ≥3.37 mmol/L (130 mg/dl) [19].

### Statistical analysis

Due to the lack of relevant prior data on Chinese occupation populations for a formal estimate of sample size, as a pilot, there no sample size was calculated. Continuous variables with normal distribution were represented by means±standard deviation. The independent-sample *t*-test was used to compare the differences between baseline continuous variables, and the chi-square test was used to compare the differences of detection rates of diseases before and after intervention. The changes of outcome variables with respect to treatment effects were tested using the mixed methods with paired-samples *t-test* and multivariate analysis. All analyses were conducted using SPSS 21.0 statistical software. Significance level was set at a two-tailed alpha of 0.05 for all tests.

## Results

### General characteristics of the participants

**Fig 1:**
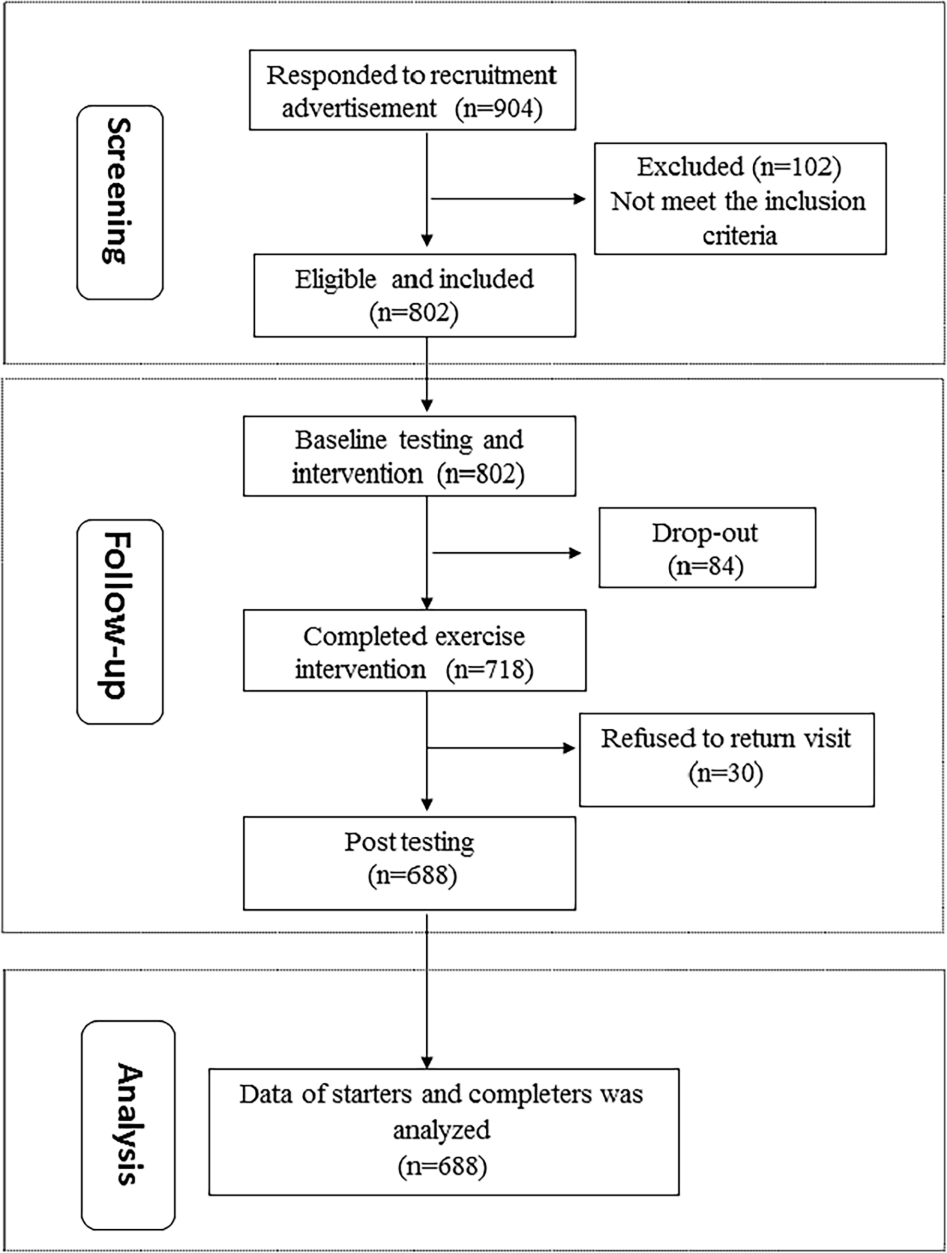
The changes in outcome measures after 3-month walking intervention for different BMI populations.

Among 904 interested occupational populations, 802 were eligible and included in one study arm (Fig 1). Of these, 718 completed the exercise program with 10.5% attrition. In addition, 688 participants who completed all programs within the study protocol with an overall completion rate of 85.8%, were analyzed.

For overweight and obese participants, body weight, WC, SBP, DBP, and TG were significantly higher in men than in women; however, BF% and HDL-C were significantly lower in men (p < 0.05). Among normal-weight participants, men had higher body weight, BMI, WC, SBP, DBP, FSG and TG but lower BF% and HDL-C (p <0.05) than women. For the underweight, men had higher body weight, WC and SBP but lower BF% and TC (p <0.05) (Table 1).

**Table 1:** Baseline characteristics of 802 subjects stratified different BMIs. Walking intervention clinical trial in China, 2015–2016.

Indicators	BMI≥24kg/m^2^			18.5≤BMI<24kg/m^2^			BMI < 18.5kg/m^2^		
	Male, M±SD	Female, M±SD	Between sex p-value	Male, M±SD	Female, M±SD	Between sex p-value	Male, M±SD	Female, M±SD	Between sex p-value
N	227	133	—	162	236	—	9	35	—
age(yrs)	37.2±9.6	41.1±9.5	<0.001	34.5±9.3	36.1±9.6	0.10	27.3±4.2	29.0±6.7	0.49
Height(cm)	172.9±5.7	159.9±5.0	<0.001	172.3±5.2	160.8±5.2	<0.001	174.8±4.6	160.2±4.5	<0.001
weight(kg)	82.9±12.1	70.6±10.2	<0.001	65.9±5.9	55.2±5.3	<0.001	55.1±3.9	45.6±3.4	<0.001
BMI(kg/m^2^)	27.7±3.4	27.6±3.3	0.87	22.2±1.4	21.3±1.5	<0.001	18.0±0.4	17.7±0.7	0.24
WC(cm)	94.8±9.6	88.9±8.9	<0.001	81.9±7.1	73.7±5.9	<0.001	71.6±6.8	66.0±4.2	0.003
BF% (%)	26.7±4.6	37.1±5.8	<0.001	20.1±4.5	27.2±4.9	<0.001	14.3±4.2	20.0±3.0	<0.001

Abbreviation: BMI, body mass index; WC, waist circumference; BF %, body fat percentage.

### Intervention effects of different BMI groups

#### Effects in overweight and obese participants

Body weight/BMI, WC, and BF% decreased by 2.2%, 1.8% and 3.3% after intervention, respectively (p <0.001), and SBP, DBP, FSG, and TC decreased by 3.3 mmHg, 3.2 mmHg, 0.11 mmol/L and 0.13 mmol/L, respectively (p < 0.05). There was no significant change in TG or HDL-C. The lifestyle behavior scores improved after intervention (p<0.05). Further analysis stratified by sex, revealed that body weight, WC, TG, and TC of men decreased more than that of women (p < 0.05), and no significant gender differences were observed with other outcomes (Table 2).

**Table 2:** Baseline and changes in outcomes variable at 3 months for different BMIs’ populations.

Indicators	Time	Male		Female		Between sex p-value
		Mean(95%CI)	Within group p-value	Mean(95%CI)	Within group p-value	
BMI≥24kg/m^2^						
Number		195		110		
Weight (kg)	Baseline	81.7(80.1, 83.2)	Ref	69.3(67.4,71.1)	Ref	<0.001
	Δat month 3	-2.0(-2.4, -1.5)	<0.001	-1.2(-1.7,-0.6)	<0.001	0.04
BMI(kg/m^2^)	Baseline	27.3(26.9, 27.7)	Ref	27.2(26.6,27.9)	Ref	0.84
	Δat month 3	-0.6(-0.8, -0.5)	<0.001	-0.4(-0.7,-0.2)	<0.001	0.14
WC(cm)	Baseline	94.5(93.1, 95.9)	Ref	87.9(86.5,90.0)	Ref	<0.001
	Δat month 3	-2.3(-3.0, -1.7)	<0.001	-0.9(-1.7,-0.2)	0.01	0.001
%BF (%)	Baseline	26.6(25.8, 27.4)	Ref	36.6(35.8,38.0)	Ref	<0.001
	Δat month 3	-1.1(-1.1, -0.7)	<0.001	-0.9(-1.7,-0.7)	<0.001	0.58
SBP (mmHg)	Baseline	125.4(123.6,127.2)	Ref	120.0(117.3,122.6)	Ref	<0.001
	Δat month 3	-2.8(-4.3,-1.3)	<0.001	-4.1(-6.1,-2.1)	<0.001	0.50
DBP(mmHg)	Baseline	83.0(81.5, 84.5)	Ref	77.9(76.0,79.9)	Ref	<0.001
	Δat month 3	-3.5(-4.7,-2.2)	<0.001	-2.8(-4.5,-1.0)	0.002	0.29
FSG(mmol/L)	Baseline	5.38(5.26, 5.49)	Ref	5.33(5.18,5.49)	Ref	0.66
	Δat month 3	-0.16(-0.28,-0.05)	0.005	-0.02(-0.13,0.09)	0.74	0.07
TG(mmol/L)	Baseline	2.16(1.90,2.42)	Ref	1.51(1.33,1.69)	Ref	<0.001
	Δat month 3	-0.19(-0.36,-0.01)	0.04	0.10(-0.04,0.25)	0.17	0.03
TC(mmol/L)	Baseline	4.88(4.68,5.07)	Ref	4.67(4.50,4.84)	Ref	0.12
	Δat month 3	-0.22(-0.39,-0.06)	0.009	0.02(-0.12,0.16)	0.62	0.04
LDL-C(mmol/L)	Baseline	2.66(2.45,2.86)	Ref	2.65(2.49,2.81)	Ref	0.95
	Δat month 3	0.03(-0.13,0.19)	0.75	0.13(0.01,0.25)	0.04	0.38
HDL-C(mmol/L)	Baseline	1.20(1.23,1.27)	Ref	1.34(1.27,1.41)	Ref	0.005
	Δat month 3	0.02(-0.05,0.08)	0.66	0.004(-0.05,0.06)	0.89	0.82
Lifestyle scores	Baseline	6.1(5.8, 6.3)	Ref	7.2(7.0, 7.5)	Ref	<0.001
	Δat month 3	0.5(0.3, 0.7)	<0.001	0.5(0.3, 0.3)	<0.001	0.76
18.5≤BMI<24kg/m^2^						
Number		131		215		
Weight (kg)	Baseline	65.6(64.6,66.7)	Ref	55.2(54.5,56.0)	Ref	<0.001
	Δat month 3	-0.5(-1.1,0.1)	0.10	-0.4(-0.7,-0.1)	0.01	0.84
BMI(kg/m^2^)	Baseline	22.1(21.9,22.4)	Ref	21.3(21.1,21.5)	Ref	<0.001
	Δat month 3	-0.17(-0.36,0.03)	0.10	-0.16(-0.28,-0.04)	0.01	0.78
WC(cm)	Baseline	81.5(80.2,82.8)	Ref	73.8(72.9,74.6)	Ref	<0.001
	Δat month 3	-0.8(-1.60,0.04)	0.06	-0.5(-1.0,0.1)	0.10	0.46
%BF (%)	Baseline	20.4(19.4,21.5)	Ref	27.3(26.7,27.9)	Ref	<0.001
	Δat month 3	-0.7(-1.45,-0.02)	0.04	-0.6(-1.09,-0.07)	0.03	0.73
SBP (mmHg)	Baseline	118.7(116.7,120.6)	Ref	109.0(107.4,110.6)	Ref	<0.001
	Δat month 3	-0.0(-1.8,1.7)	0.99	-1.4(-3.0,0.1)	0.07	0.24
DBP(mmHg)	Baseline	76.6(74.9,78.3)	Ref	70.9(69.6,72.1)	Ref	<0.001
	Δat month 3	-0.6(-2.2,1.0)	0.47	-1.3(-2.5, 0.0)	0.04	0.52
FSG(mmol/L)	Baseline	5.13(5.02, 5.24)	Ref	4.95(4.87, 5.02)	Ref	0.007
	Δat month 3	-0.15(-0.26, -0.05)	0.04	-0.10(-0.18, -0.02)	0.02	0.41
TG(mmol/L)	Baseline	1.31(1.14, 1.48)	Ref	1.02(0.94, 1.09)	Ref	0.002
	Δat month 3	0.15(-0.01, 0.31)	0.07	0.06(-0.02, 0.14)	0.11	0.35
TC(mmol/L)	Baseline	4.36(4.20, 4.53)	Ref	4.33(4.19, 4.47)	Ref	0.75
	Δat month 3	-0.05(-0.22, 0.12)	0.58	0.01(-0.10, 0.12)	0.87	0.58
LDL-C(mmol/L)	Baseline	2.19(2.02, 2.36)	Ref	2.23(2.10, 2.35)	Ref	0.74
	Δat month 3	0.19(0.05, 0.32)	0.008	0.06(-0.06, 0.18)	0.36	0.17
HDL-C(mmol/L)	Baseline	1.38(1.31, 1.45)	Ref	1.46(1.41, 1.51)	Ref	0.05
	Δat month 3	0.04(-0.01,0.09)	0.13	0.04(-0.02, 0.10)	0.18	0.97
Lifestyle scores	Baseline	5.1(4.6, 5.6)	Ref	7.0(6.7,7.2)	Ref	<0.001
	Δat month 3	1.1(0.7, 1.5)	<0.001	0.7(0.4,0.9)	<0.001	0.06
BMI < 18.5kg/m^2^						
Number		5		32		
Weight (kg)	Baseline	55.6(49.3, 61.9)	Ref	45.5(44.3, 46.8)	Ref	<0.001
	Δat month 3	1.1(-1.6, 3.8)	0.34	0.4(0.0, 0.8)	0.05	0.27
BMI(kg/m^2^)	Baseline	18.2(17.7, 18.8)	Ref	17.7(17.5, 17.9)	Ref	0.19
	Δat month 3	0.3(-0.6, 1.2)	0.42	0.2(0.0, 0.3)	0.04	0.49
WC(cm)	Baseline	70.7(66.8, 74.7)	Ref	66.0(64.5, 67.5)	Ref	0.02
	Δat month 3	1.2(-1.8, 4.2)	0.32	0.1(-1.0, 1.2)	0.86	0.45
%BF (%)	Baseline	15.4(9.3, 21.6)	Ref	20.4(19.4, 21.4)	Ref	0.002
	Δat month 3	-0.2(-1.3, 0.9)	0.65	0.2(-0.5, 0.9)	0.51	0.61
SBP (mmHg)	Baseline	115.0(109.3, 120.7)	Ref	106.5(103.9,109.1)	Ref	0.02
	Δat month 3	-3.6(-15.8, 8.6)	0.46	-0.3(-2.9,2.2)	0.78	0.37
DBP(mmHg)	Baseline	76.8(65.3, 88.3)	Ref	70.2(67.5, 72.9)	Ref	0.09
	Δat month 3	-3.0(-11.2, 5.3)	0.37	-1.2(-3.4, 1.0)	0.27	0.54
FSG(mmol/L)	Baseline	4.65(4.16, 5.14)	Ref	4.87(4.67, 5.06)	Ref	0.40
	Δat month 3	0.30(-0.09, 0.69)	0.09	-0.11(-0.28, 0.07)	0.23	0.10
TG(mmol/L)	Baseline	0.87(0.49, 1.25)	Ref	0.96(0.62, 1.31)	Ref	0.81
	Δat month 3	0.11(-0.12, 0.34)	0.26	0.08(-0.11,0.28)	0.39	0.90
TC(mmol/L)	Baseline	3.19(1.48, 4.90)	Ref	4.13(3.84, 4.43)	Ref	0.03
	Δat month 3	0.93(-0.65, 2.50)	0.18	0.20(-0.10, 0.50)	0.19	0.28
LDL-C(mmol/L)	Baseline	1.90(1.08, 2.71)	Ref	2.24(1.89, 2.58)	Ref	0.40
	Δat month 3	0.36(-0.23, 0.94)	0.17	0.13(-0.14, 0.39)	0.34	0.46
HDL-C(mmol/L)	Baseline	1.13(0.90, 1.36)	Ref	1.68(1.32, 2.04)	Ref	0.19
	Δat month 3	0.07(-0.20, 0.35)	0.47	-0.02(-0.12, 0.08)	0.66	0.42
Lifestyle scores	Baseline	5.7(3.2, 8.2)	Ref	7.1(6.7, 7.5)	Ref	0.19
	Δat month 3	0.5(-0.1, 1.1)	0.46	0.4(0.0, 0.7)	0.66	0.77

Abbreviation: BMI, body mass index; WC, waist circumference; SBP, systolic blood pressure; DBP, diastolic blood pressure; BF %, body fat percentage; FSG, fasting serum glucose; TG, triglyceride; TC, total cholesterol; LDL-C, low density lipoprotein-cholesterol; HDL-C, high density lipoprotein-cholesterol.

**Fig 2:**
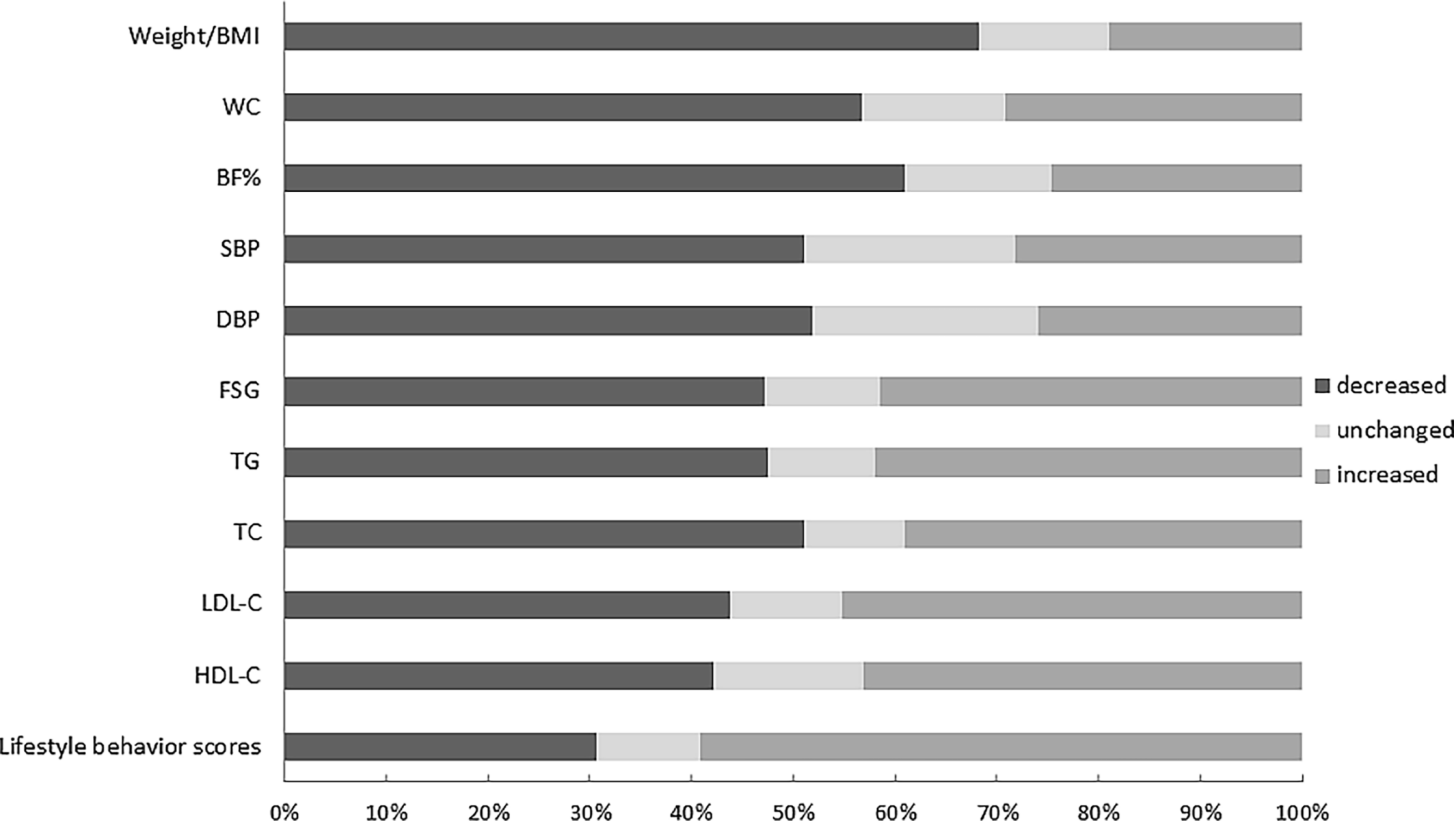
The proportion of changing state after 3-month intervention among the overweight and obesity group. Abbreviation: BMI, body mass index; WC, waist circumference; SBP, systolic blood pressure; DBP, diastolic blood pressure; BF %, body fat percentage; FSG, fasting serum glucose; TG, triglyceride; TC, total cholesterol; LDL-C, low density lipoprotein-cholesterol; HDL-C, high density lipoprotein-cholesterol.

In addition, we also calculated the proportion of participants whose parameters increased, remained unchanged, or decreased after intervention. More than 50% of the overweight/obese participants experienced reduction in body weight/BMI, WC, BF%, SBP, DBP, and TC. Majority of the participants demonstrated a decrease in FSG (47.2%) and TG (47.5%). Approximately, 59% of the participants showed improvement in lifestyle behavior (Fig 2).

In addtion, 68.2% (208/305) of overweight/obese participants experienced weight loss after intervention with an average reduction of 3.5% (2.8 kg) (p < 0.001). With respect to overweight and obesity, 13.8% (42/305) of participants lost more than 5% of their body weight. Futhermore, 20.2% (42/208) of those who experienced losing weight got success in body weight (lost ≥5% of their body weight at baseline). Ten participants had severe obesity (BMI ≥35 kg/m^2^) at baseline, with a mean weight of 111.6 kg (±18.8), BMI of 39.2 kg/m^2^ (±3.6), WC of 120.2 cm (±13.1), BF% of 44.3% (±6.8), and SBP/DBP of 129.3 mmHg (±13.8)/88.7 mmHg (±6.3). At the end of the 3-month study period, the body weight (or BMI), WC, SBP, and DBP decreased by 4.7%, 3.6%, 3.7%, and 8.1%, respectively. Decreases in body weight (or BMI), BF%, and DBP were statistically significant (p <0.05). When stratified by age, subjects aged 18–29 years experienced the most weight loss (4.6%).

#### Changes in the normal-weight group and underweight group

Body weight/BMI, WC, BF%, DBP, and FSG were significantly reduced by 0.7%, 0.8%, 2.5%, 1.4%, 2.4%, respectively, in normal-weight participants after intervention (p < 0.05). In addition, lifestyle behavior scores significantly improved (p < 0.001). There was no significant gender difference for all outcomes (Table 2).

Body weight (or BMI) of underweight participants increased by 1% after the 3-month intervention, with significant average increases of 0.5 kg in body weight and 0.18 kg/m^2^ in BMI; lifestyle behavior scores significantly improved (p < 0.05). There were no significant changes in other outcomes with no significant gender difference.

### Effects of intervention on hypertension, hyperglycemia, and hyperlipidemia

The detection rate of hypertension decreased from 14.5% at baseline to 9.3% after intervention (p < 0.001). After the 3-month intervention period, no significant changes in detection rates were observed in hyperglycemia and hyperlipidemia. The normal recovery rate of hypertension and hyperglycemia were 56% and 56.5%, respectively (Table 3).

**Table 3:** Detection rates in pre-, post-intervention and normal recovery rates of hypertension, hyperglycemia, and hyperlipidemia.

outcome	N	abnormal detection rate		normal recovery rate(post-intervention)
		pre-intervention	post-intervention	
BP	688	100/688(14.5%)	64/688(9.3%) ***	56/100(56.0%)
FSG	610	46/610(7.5%)	41/610(6.7%)	26/46(56.5%)
TG	567	134/567(23.6%)	144/567(25.4%)	40/134(29.9%)
TC	566	122/566(21.6%)	129/566(22.8%)	40/122(32.8%)
LDL-C	563	92/563(16.3%)	92/563(16.3%)	34/92(37.0%)

*P<0.05

** p<0.01

*** p<0.001(compared to the pre-intervention by chi-square test).

Abbreviation: BP, blood pressure; FSG, fasting serum glucose; TG, triglyceride; TC, total cholesterol; LDL-C, low density lipoprotein-cholesterol

## Discussion

This study showed that application of a self-monitored prescription pedometer could effectively improve the physical activity of the occupational population, reduce body weight and BMI in overweight and obese participants, improve body composition in normal-weight participants, and increase body weight and BMI in underweight participants, as well effectively control the risk factors for weight-related chronic diseases.

In this study, the combination of prescription pedometer-based exercise and IHSC helped achieve an overall compliance rate of 89.5% in exercise intervention. The 14.2% rate of attrition was lower than that observed with traditional lifestyle intervention for weight management (approximately 20%).[20] This is similar to the attrition rate in weight management using frequent text message reminders (12.7%).[21] Futhermore, we also observed that good improvement was observed after intervention in all BMI groups. Body weight and WC significantly decreased by 2.2% and 1.8%, respectively, in overweight and obese participants, and the result in men was better than that in women. For normal-weighted participants, body weight (or BMI) decreased by 0.7%, with decrease of 0.8% in WC and 2.8% in BF%. Underweight participants demonstrated a mean weight gain of 1.0% (p < 0.05). Further analysis indicated that 68.2% of the overweight/obese participants experienced weight loss, with mean weight loss of 3.5% (p < 0.001), and 20.9% of them lost ≥5% body weight. This method could also yield good weight loss results with no adverse effects for severe obesity (BMI ≥35 kg/m^2^).

Several network and new technology-based studies on weight management have shown that 3-month lifestyle interventions reduced body weight by 2%-4% and markedly improved health outcomes [22,23,24,25,26]. Our results were consistent with those of the abovementioned studies on weight loss and health benefits. However, limitations of the previous internet-supported interventions included a higher staff burden (e.g., researchers vs. participants of 1: 10 to 1:20), more frequent internet-based health lessons (at least once a week), or requirement of face-to-face communication and frequent reminding (daily SMS or e-mail reminding), etc. In comparison, the staff burden of 1: 100 or more and remote management and supervision in our study were more economical, more convenient and more suitable for intervention in large populations. This intervention is simple, feasible and is effective in risk factor control, particularly for cardiovascular disease. Walking is a major part of our daily life and for the overweight population, moderate and vigorous walking is a safer, easier and more effective strategy of weight management [27,28,29,30]. This intervention is based on walking, with personalized modifications of intensity, duration, and frequency of the exercise, depending on the general health and lifestyle of each participant to achieve greater benefits and compliance. Daily exercise duration was divided into three parts, which were performed continuously or at separate times. This is particularly advantageous to the working population because they can schedule the exercises according to their available spare time. Moreover, the exercises can be performed at any place.

Futermore, the intervention achieved a clinically significant improvement in the risk factors of cardiovascular disease and metabolic syndrome, particularly in blood pressure and blood glucose. For both the overweight/obese group and the normal-weight group, the BP, FSG and BF% decreased after the intervention (1%-4%). The decreases in the overweight and obese participants were greater than those in normal-weight participants. The incidence of hypertension was also significantly decreased after the intervention. This was consistent with the improvement in cardiovascular risk factors through Internet-based lifestyle interventions [31]. Compared to the studies on diabetes-related lifestyle intervention in Daqing (51% recovery rate of hyperglycemia), the United States (58%) and Finland (43%)[32,33,34], our study achieved a 56.5% recovery rate of hyperglycemia with no dietary restriction and continuous management, indicating that the intervention program in our study has potential for diabetes risk management. Additionally, the recovery rates for hypertion, high TG, high TC, and high LDL-C were approximately 56.0%, 29.9%, 32.8%, and 37.0%, respectively. These findings demonstrated the effectiveness of this intervention program not only in weight control but also in chronic disease risk control. The effect of improvement in the level of blood lipids, however, is low, which might be because of the less intensive dietary intervention. In this study, dietary intervention was delivered through a one-time guidance, with no follow-up or dietary restriction. Nevertheless, a significant improvement was achieved in the control of abdominal obesity, hypertension, hyperglycemia, and hyperlipidemia, which are major risk factors for cardiovascular and cerebrovascular diseases and also a variety of other chronic diseases[35,36,37,38], indicating that this intervention has a promising potential for effective health management.

Another innovation of this intervention is that the exercise prescription has to be downloaded to the pedometer for self-monitoring. Only when 80% or more of the tasks in the prescription of the previous month was completed properly would the prescription be upgraded (higher intensity and/or longer duration of exercise) in the following month. Otherwise, the same prescription would be continued. This method of increasing the amount of exercise (including exercise intensity and exercise time) ensures the safety of the subject’s exercise process, thus reducing the incidence of motor trauma. Using this "one vs. many" remote monitoring and management model, one management staff is able to monitor and guide as many as hundreds or even thousands of participants, and thus reduce staff burden and time consumption.

Our study has several limitations. Firstly, only short-term (3 months) effects were observed. A longer follow-up is required to evaluate the long-term effectiveness of this intervention. Secondly, the dietary part of the intervention needs to be developed and improved futher. Thirdly, the relationship between the level of exercise and the intervention effects remains to be analyzed.

## Conclusions

The combined uses of the exercise prescription pedometer-based and Internet-based Health System Center to improve the level of physical activity can effectively manage the weight of occupational population and can significantly improve risk factors of weight-related diseases. This intervention is both simple to implement and promote public health in a feasible and effective manner. It is expected to play an important role in weight management and health promotion, as well as control and management of risk factors of chronic disease.

## Supporting information

S1 File TREND.pdf. (PDF)

S2 File Protocol_in Chinese.pdf. (PDF)

S3 File Protocol_V-01.pdf. (PDF)

S4 File ORIGINAL DATA.xlsx. (XLSX)
