# Supplementary material for: A novel prescription pedometer-assisted walking intervention and weight management for Chinese occupational population
Source: PLoS One. 2018 Jan 11;13(1):e0190848. doi: 10.1371/journal.pone.0190848 (PMC5764333; doi:10.1371/journal.pone.0190848)
Supplement: S2 File — (PDF) [file pone.0190848.s002.pdf]

研究题目：利用 IT 技术和处方计步器对成人肥胖量化运动减肥的研究

日期：2014.10.10　　　　　　版本号：PKU IRB_V-01

课题负责人：常翠青　　　　单位：北医三院　　　　科室：运动医学

签名页

已经阅读和批准此文件提出的方案，并对方案相关内容予以认同。签名者应包括所有参与方案制定人员。

保密声明。

姓名	职务	职称	任职部门	签名
常翠青	室主任	研究员	运动医学院研究所	
艾华		研究员	运动医学院研究所	
陈秀云	护士长	副主任护士	运动医学院研究所	
陈志民		主管技师	运动医学院研究所	
谢岚		主管技师	运动医学院研究所	
吕祎然		硕士生	运动医学院研究所	
玉应香		硕士生	运动医学院研究所	

研究摘要

题目	利用 IT 技术和处方计步器对成人肥胖量化运动减肥的研究
研究目的	利用 IT 技术管理平台和处方计步器,探讨适合中国成人体重管理、超重和肥胖人群体重控制的最佳有效运动量；观察生活方式干预与肥胖基因（PLIN 基因）相互作用对中国成年人体重管理的影响，探讨个体化运动减肥处方。
研究概要	700 名 18-65 岁中国成年人肥胖、超重、正常体重以及低体重者，均给予个体化的运动处方和膳食处方。将运动处方下载至处方计步器中，同时进行健康教育，周期为 6 个月。干预前、中、后分别进行体格测量、血生化检测及 PLIN 基因 SNPs 检测,以便探讨适合中国成人体重控制的最佳有效运动量，及其与 PLIN 基因相互作用对体重管理的影响。
实验设计	自身对照开放性干预研究。
对象	700 名 18-65 岁中国成年人超重或肥胖者，或有体重管理意愿者，符合本研究的入组标准，并签署知情同意书。
干预措施	以中等强度的健步走为主，辅以肌肉力量训练和柔韧性训练，30-90min/天，≥3 天/周，前 3 个月运动量逐月递增；限能量平衡膳食，脂肪 20-25%、碳水化合物 50-60%、蛋白质 15-20%；健康教育，包括科学运动、平衡膳食等内容。
主要观察指标	主要指标：身高、体重、体脂、腰围、血压；次要指标：血脂、血糖及相关指标、炎性因子、脂肪因子、PLIN SNPs。
干预周期	3 个月和 6 个月
项目负责人	运动医学研究所 常翠青研究员

目录

签名页 **2**

研究摘要 **3**

目录 **4**

正文 **5**

1 研究背景 5
2 研究目的 6
3 研究设计 7
3.1 研究对象 7
3.2 试验分组 7
3.3 研究方案 7
3.4 随访计划 8
3.5 主要测量指标或结局指标的选择和确认 9
3.6 样本量的计算和推理 9
4 数据管理 10
4.1 数据录入 10
4.2 数据核查和管理的内容和方式 10
4.3 数据存档 10
5 统计分析 10
6 安全性评价 11
6.1 不良事件（AE）和严重不良事件（SAE） 11
6.2 不良事件的报告 11
7 受试者保护 11
8 研究管理 11
8.1 方案修改 11
8.2 提前终止 12
9 流程与进度 12
10 参考文献 12

正文

1 研究背景

作为全球经济发展最快的国家，我国在过去短短的三十年中经历了西方国家需要一两百年才完成的膳食和生活方式转型。以高能量、高脂肪和高动物性食物为特征的膳食结构的改变，加上体力活动的缺乏，导致了肥胖相关的慢性代谢性疾病的发病率井喷式增长。2002 年我国 18 岁及以上成人的超重和肥胖率分别为 22.8% 和 7.1%，较 1992 年分别增长了 40.7% 和 97.2%[1]。最近的全国疾病监测 (DSPs) 的数据显示：2010 年我国成人的超重和肥胖率已分别达到了 30.6% 和 12.0%。由于超重基数大, 预计今后肥胖患病率将以较大幅度增长。研究表明：肥胖可以增加 2 型糖尿病、胆囊炎、高血脂和胰岛素抵抗 3 倍以上的患病风险；增加冠心病、高尿酸血症、痛风、骨关节炎等 2~3 倍的患病风险。除此之外，超重和肥胖也会引发一系列社会和心理问题。

研究表明，肥胖者适量减重（5%—10%）能够降低肥胖相关疾病的发生风险[2]。在现有的体重干预手段中，合理膳食和运动是控制体重的有效方法。 8 个基于 RCT 的 meta 分析显示，改善膳食结构能带来适宜的、具有临床意义的体重减少；4 个基于 RCT 的 meta 分析显示，体力活动干预能带来活动习惯的改变，改善心肺功能[3]。但是，随着肥胖发生率逐步上升，肥胖人群迅速扩大；生活方式干预虽然有效，但因依从性差、针对个体或小单位群体进行干预覆盖面小，常常是费时、费力、耗财，对整个肥胖的控制收效甚微。近年由于 IT 网络通讯技术的发展和普及，给体重管理和减肥方法提供了新的思路。IT 技术应用广泛，几乎渗透每个领域，其用户庞大、受众广，且能实现与大样本人群的实时沟通，在节约了大量人力资源和费用的同时，能实现对受试者进行长期随访、监督，进行动态管理。前期我实验室参与的一项与美国杜克大学合作的项目，利用每天发送手机短信，用普通计步器作为监督工具，在北京进行的为期 6 个月的临床随机对照研究显示，利用手机短信辅助的生活方式干预对降低超重者的体重、腰围、血压等指标有显著效果[4]。但普通计步器只能机械的记录下包括日常活动的总步数，无法提示受试者以适宜的强度和有效的运动量进行运动，因此，如何客观地监督人们在有限的时间内进行科学有效的运动，值得研究。

肥胖的发生受多种因素和基因的影响。生活方式干预并非对每个人有效。除了个体依从性的差异之外，还存在个体基因水平的差异。PLIN 基因是肥胖的候选基因之一，编码的脂肪细胞相关蛋白 Perilipin 是包被在脂肪细胞内脂滴表面的一种可磷酸化蛋白。人类的 PLIN 基因位于 15q26.1，邻近肥胖、糖尿病和高甘油三酯血症的易感位点[5]。研究表明，PLIN 基因 SNP 与肥胖风险及血糖、脂代谢有关，并且对饮食干预及运动干预后肥胖人群的减肥效果有所影响，不同基因型经过干预后的减肥效果也具有差异。目前，国内外已发现的人 PLIN 基因多态性有 218 个，其中有意义的主要是 PLIN1（6209T>C）、PLIN3（17071A>T）、PLIN4（11482G>A）、PLIN5（13041A>G）、PLIN6（14995A>T）及 PLIN7（13042A>G）。本实验室前期就 PLIN 基因单核苷酸多态性在肥胖人群中的分布、以及与减肥效果的关系进行了初步研究。结果显示，在中国汉族成人肥胖受试者中，PLIN1 罕见基因 C 占多数，C 表型可能与肥胖风险相关，PLIN4 以常见基因 G 表型为主，罕见基因 A 表型可能与低 BMI 和低肥胖风险相关；PLIN6 以常见基因 A 表型多见，罕见基因 T 表型可能与成年女性低 BMI 和低肥胖风险相关[6]。因此，研究 PLIN 基因 SNP 在人群中的分布及其与生活方式相互作用，对进行肥胖个体化治疗具有重要作用。

本研究拟通过建立生活方式干预肥胖 IT 管理平台及处方计步器应用，观察这些技术在肥胖防治中的作用，探讨体重管理和减肥的最佳有效运动量和个体化运动处方，为实时进行大人群体重管理和肥胖干预及个体化治疗技术提供科学依据。

2 研究目的

主要目的：利用 IT 技术管理平台和处方计步器，探讨适合中国成人体重管理、超重和肥胖人群体重控制的最佳有效运动量；

次要目的： 观察生活方式干预与肥胖基因（PLIN 基因）相互作用对中国成年人体重管理的影响，探讨个体化运动减肥处方。

3 研究设计

3.1 研究对象

本试验的研究对象为 18-65 岁中国成年体重超重和肥胖者、以及有体重管理意向的志愿者。体重判断标准参考“中国成人超重和肥胖筛查体重指数标准”，BMI < 18.5 kg/m^2 为低体重，BMI 为 18.5～23.9kg/m^2 为正常体重，BMI≥24kg/m^2 为超重，BMI≥28kg/m^2 为肥胖；.排除标准包括：继发性肥胖以及有心、肺、肝、肾等重要器质病变、身体发育异常残缺畸形、怀孕或计划在 10 个月内怀孕、哺乳期妇女、血压超过 180/110mmHg 或出现高血压并发症、空腹血糖超过 16.7mmol/L 或出现糖尿病并发症、正在服用减肥药物及任何会导致体重增加的药物或激素、经历过或者计划进行减肥手术。

3.2 研究设计

本研究为自身对照开放性干预研究。拟以职业人群和企业机构为单位，采取整群抽样和招募广告结合方法，招募志愿者。

3.3 研究方案

1. 建立生活方式干预肥胖 IT 技术平台

（1）医学方案的制定

首先采集受试者的基本信息，根据信息进行评估及人群分类（性别、年龄、BMI 等），而后针对不同人群目前身体活动水平和心肺功能，分别制定两个阶段的运动处方与膳食处方，每个阶段又分三个水平，并定期评价各处方完成情况。

（2）IT 平台的建设

与得实信息（北京）科技有限公司合作，严格按照医学方案，应用电脑编程技术，将完整的医学方案以健康管理系统应用软件的形式呈现出来，建立生活方式干预健康管理 IT 技术平台。

2. 应用 IT 技术平台进行体重管理和肥胖干预研究

根据医学评估结果，给予个性化运动处方和营养指导，并将个性化运动处方下载到处方计步器。干预周期为 3～6 个月。

干预方案：

（1）运动干预：有氧运动为主，抗阻运动为辅，适当柔韧性运动。运动量逐月递增。

运动强度：中等强度，以步/分钟呈现；

运动方式：以健走为主，辅以肌肉力量训练（抗阻运动）和柔韧度训练；

运动时间：30-90min/天，具体的时间以个性化的运动处方为准。

运动频率：≥3 天/周

根据受试者初始时身体活动水平，制定个体化运动处方，并将运动处方下载至计步器中，提醒受试者按处方要求的强度和时间进行运动。若处方完成率较好（≥80%），则运动强度和运动量在前 3 个月内按梯度逐月递增。

（2）膳食处方：控能量平衡膳食

控能量平衡膳食：根据标准体重、身体活动水平确定摄入总能量，给予膳食处方和食谱示例。三大营养素供能比：脂肪 20-25%、碳水化合物 50-60%、蛋白质 15-20%；

（3）健康教育：平衡膳食、合理营养、健康膳食选择、科学运动、安全运动，等：干预前、干预第 3 个月，共 2 次；

（4）定期、实时监测：利用计步器对运动量进行实时监测，利用调查问卷对抗阻运动和膳食进行定期监测，1 次/ 3 个月。针对个人随时提醒、调整饮食结构及行为。

3.4 随访计划

时期	入选期	入组后	M1		M2		M3		M4		M5		M6	
时间	-8—-4 周	-4—0 周	第 1-3 周	第 4 周	第 5-7 周	第 8 周	第 9-11 周	第 12 周	第 13-15 周	第 16 周	第 17-19 周	第 20 周	第 21-23 周	第 24 周
签署知情同意	√													
入选和排除标准	√													
基本资料		√												
体格测量		√						√						√
生活方式调查		√						√						√
膳食调查		√						√						√
身体活动水平调查（长）		√						√						√

身体活动水平调查（短）		√						√						√
一周运动监测情况			√	√	√	√	√	√	√	√	√	√	√	√
健康教育		√						√						
PLIN 基因 SNPs		√												
血压、血糖		√						√						√
血生化		√						√						√
不良事件监测		√	√	√	√	√	√	√	√	√	√	√	√	√
合并用药		√	√	√	√	√	√	√	√	√	√	√	√	√

3.5 主要测量指标或结局指标的选择和确认

测量指标包含基线、干预 3 个月（中期）、干预 6 个月时的以下内容：

主要指标：

体格测量指标：身高、体重、体脂（体成分分析仪），腰围（参照 WHO 腰围测定方法）；BMI，腰围/身高。

次要指标：

生活方式、膳食结构和身体活动水平：生活方式调查问卷、食物频率调查问卷、IPAQ 问卷；

血压：汞立式血压计；上臂式电子血压计(欧姆龙)

空腹血糖、OGTT 和血脂：全自动血生化分析仪；

血清胰岛素：ELISA 法；

血浆超敏 CRP、脂联素、瘦素：ELISA 法；

PLIN1、PLIN4、PLIN6 位点单核苷酸多态性：一代测序法。

3.6 样本量的计算和推理

根据公式 $N=2\times[(Z\alpha+Z\beta)\times\delta/d]^2$ 及本实验室前期研究得出 SNP 在汉族肥胖成人群中的分布及失访率(20%)，假定双侧检验 $\alpha=0.05$，检验效能 80%，计算得出每组需要样本 348 例，两组体重超重、肥胖与否，则需入选约 700 例受试者以保证试验质量。

4 数据管理

4.1 数据录入

受试者的基本信息和相应调查问卷在基线时通过 IT 平台录入及保存。其他生化指标和基因型信息将由测试执行者在测试完成后及时、准确的录入相应的数据库。运动量数据由受试者将计步器中的数据通过 USB 接口上传至网络，研究专业人员通过 IT 技术管理平台定期对上传数据进行收集、整理、评估。

4.2 数据核查和管理的内容和方式

研究人员完成信息采集和记录后，将各自数据库交予数据管理员进行审查、整合，由数据管理员写出检查报告，其内容包括受试者完成情况（含失访者信息）、入选/排除标准检查、完整性检查、不良事件检查等。

4.3 数据存档

调查问卷在按要求完成数据录入和核查后，按受试者编号顺序归档保存，以备查考。电子数据文件包括数据库、分析程序、分析结果等应分类保存，并有多个备份保存于不同磁盘或记录介质，防止损坏。

5 统计分析

应用利益变量单因素分析检验基线时的组间差异、离群值、和分布假设；基因分布采用 Hardy-Weinberg 平衡检验；基因连锁采用 Haploview 软件进行 Linkage-Disequilibrium 分析；体格测量指标和血生化指标会根据情况使用 t 检验、方差分析和非参数检验来检测各结果的组间差异；采用基线数据结转填补法处理缺失数据以进行 ITT 分析。所有统计分析均采用双侧检验，$P<0.05$ 被认为所检验指标的差别有统计学意义。

6 安全性评价

6.1 不良事件（AE）和严重不良事件（SAE）

本研究可能发生的不良事件主要是指由于运动不当造成的运动损伤，但对受试者在干预期间中出现的任何导致不能继续参与试验的情况都应仔细记录并及时报告。

6.2 不良事件的报告

如发生任何不良事件，无论是否与研究干预有关，也无论是否已实施干预操作，均必须在发生/获知 24 小时内通过电话/短信通知研究者。本研究属于开放、远程监督方式的管理，若受试者未向研究者报告不良事件，则默认为无不良事件发生。

7 受试者保护

本实验方案和知情同意书获北京大学第三医院生物医学伦理委员会批准后，并在招募受试者期间，研究人员向受试者充分说明和解释该研究背景、性质、意义、步骤、受益、风险、补偿、退出等事项后，受试者自愿同意并签署知情同意书后，进行研究。研究期间发生任何不良事件，按要求及时报告，并积极给予妥善处理。

8 研究管理

8.1 方案修改

可能由于监查人员发现不遵守入选标准的现象持续存在导致了变化，也可能由于入选标准过于严格导致了招募人数过低，研究方案中对于这部分的变化进行的修订，考虑到会影响统计结果，可能导致样本量调整，或是对于分析计划进行的修订。

8.2 提前终止

定义提前终止研究的原则及处理办法，如：

1. 试验中发现严重安全性问题，应及时终止临床试验。
2. 试验中发现所定临床试验方案有重大失误，难以评价干预效应；或在实施中发现了严重偏差，难以评价干预效应。
3. 申请人要求终止或行政管理部门要求终止实验。

9 流程与进度

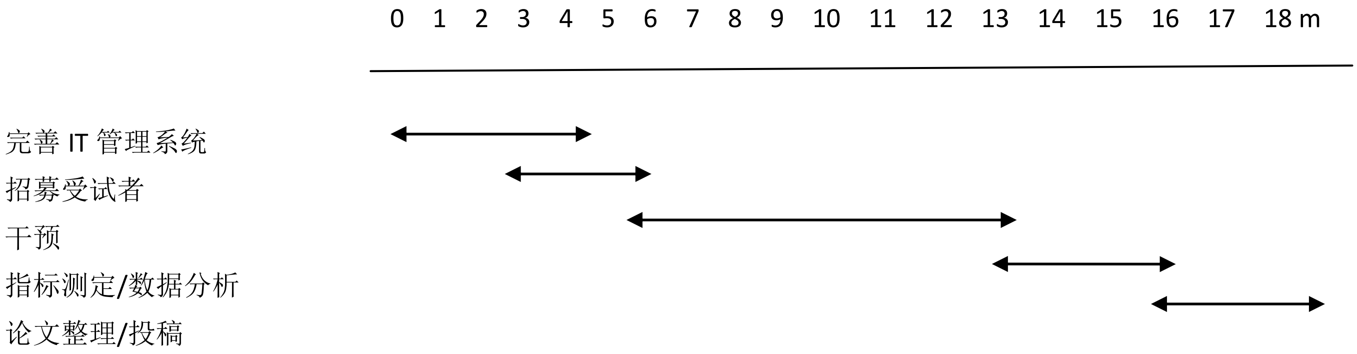

10 参考文献

[1] 林旭，黎怀星，叶兴旺，等. 环境和遗传因素与慢性代谢性疾病的人群研究. 生命科学, 2012, 24(07): 614-625.

[2] National Heart Lung and Blood Institute (NHLBI). Clinical Guidelines on the identification, evaluation, and treatment of overweight and obesity in adults–the evidence report (ClinicalGdlns). Obes Res 1998; 6: 51S–209S.

[3] Curioni CC, Lourenco PM, Long-term weight loss after diet and exercise: a systematic review. Int J Obes (Lond), 2005, 29(10): 1168-74.

[4] Lin PH, Wang Y, Levine E, et al. A text messaging-assisted randomized lifestyle

weight loss clinical trial among overweight adults in Beijing. Obesity (Silver Spring), 2014, 22(5): E29-37.

[5] Rankinen T, Zuberi A, Chaqnon YC, et al. The human obesity gene map: the 2005 update. Obesity (Silver Spring), 2006, 14(4): 529-644.

[6] 陈燕波，常翠青，黄志卓，等, PLIN 基因多态性在中国汉族成年肥胖者中的分布. 营养学报, 2011,33(01): 29-33.
