# Supplementary material for: A novel prescription pedometer-assisted walking intervention and weight management for Chinese occupational population
Source: PLoS One. 2018 Jan 11;13(1):e0190848. doi: 10.1371/journal.pone.0190848 (PMC5764333; doi:10.1371/journal.pone.0190848)
Supplement: S3 File — (PDF) [file pone.0190848.s003.pdf]

Title of Study: IT technology based and prescription pedometer assisted exercises for personalized weight management and weight loss in Chinese adults

Version:PKU IRB_V-01 Date:2014.10.10

Principal Investigator:Cuiqing Chang

Institution: Peking University Third Hospital

Department: Sports Medicine

Signature Page

The program presented in this document has been read and approved, and the relevant content of the program has been endorsed. The signer should include all participants in the program development.

Privacy Statement.

Name	Position	Technical Title	Department	Signature
Cuiqing Chang	Principal Investigator	Chief Researcher	Sports Medicine	
Hua Ai		Senior Researcher	Sports Medicine	
Xiuyun Chen		Co-chief nurse	Sports Medicine	
Zhiming Chen		Technician	Sports Medicine	
Lan Xie		Technician	Sports Medicine	
Yiran Lv		Postgraduate Student	Sports Medicine	
Yingxiang Yu		Postgraduate Student	Sports Medicine	

Synopsis

Scientific Title	IT technology based and prescription pedometer assisted exercises for personalized weight management and weight loss in Chinese adults
Objective	Main Objective: To explore the IT technology management platform-based and prescription pedometer-assisted exercise on weight management for Chinese adults, and to find the minimum effective amount of exercise on weight loss for obese adults.<br>Secondary Objective: To observe the effects of lifestyle intervention and obesity gene (PLIN gene) interaction on weight management in Chinese adults.
Study Outline	700 of Chinese adults whose age 18 to 65 years, underweight, normal weight, overweight, obesity, are given individual exercise prescription and diet prescriptions. The exercise prescription will be downloaded to the prescribing pedometer, and the health education will be carried out at the same time. The intervention period is 6 months. At the pre-, in the mid- and post- the intervention, physical measurements and blood biochemical tests will be performed respectively. Meanwhile, a PLIN SNPs test will be carried out in order to explore the optimal amount of effective exercise for Chinese adult weight control and the effect of PLIN SNPs on body weight management.
Study Design	open, self-control intervention trail
Subjects	700 of obesity, overweight adults or normal weighted adults who are willing to manage their weight or improve their lifestyle, aged 18-65 years, with written informed consent form.
Intervention	Exercise: 30-90min / day, ≥3 days / week; The intensity or the duration of exercise increased month by month at first 3 months.<br>Diet: limited energy balance diet, fat of 20-25%, Carbohydrates of 50-60%, protein of 15-20%.<br>Health education: including scientific exercise, balanced diet and so on.
Outcomes	Primary indicators: Height, body weight, body fat, waist circumference.<br>Secondary indicators: Blood pressure, serum lipid (including triglyceride, total cholesterol, low density lipoprotein cholesterin, high density lipoprotein cholesterin), fasting blood-glucose and its related indicators, inflammatory cytokines, adipocytokines and PLIN SNPs.
Intervention period	3 months and 6 months
Principal Investigator	Dr. Cuiqing Chang

Contents

SIGNATURE PAGE 2

SYNOPSIS 3

CONTENTS 4

MAIN BODY 5

1 BACKGROUND 5
2 OBJECTIVE 7
3 STUDY DESIGN 8
3.1 Subjects *8*
3.2 Study design *8*
3.3 Research procedures *8*
3.4 Follow-up Plan *10*
3.5 Outcome and its measurement *11*
3.6 Sample size calculation *12*
4 DATA MANAGEMENT 12
4.1 Data input *12*
4.2 Data verification and management methods *12*
4.3 Data archiving *12*
5 STATISTICAL ANALYSIS 13
6 SAFETY ASSESSMENT 13
6.1 Adverse events (AE) and serious adverse events (SAE) *13*
6.2 Reporting of serious adverse events *13*
7 SUBJECTS PROTECTION 14
8.1 Protocol Amendment *14*
8.2 Premature trial termination *14*
9 PROCESSES AND PROGRESS 15
10 REFERENCES 15

Main Body

1 Background

As one of the fastest growing economies in the world, China has undergone a dramatic transformation of its diet and lifestyle in the past three decades, which may take a century or two to complete in Western countries. Changes in dietary patterns characterized by high energy, high fat and high animal foods, coupled with a lack of physical activity, have led to an increase in the incidence of obesity-associated chronic metabolic diseases. In 2002, the overweight and obesity rates of Chinese adults aged 18 and above were 22.8% and 7.1% respectively, which increased by 40.7% and 97.2% compared with 1992 [1]. Recent data from the National Disease Surveillance (DSPs) showed that overweight and obesity rates in China in 2010 were 30.6% and 12.0%, respectively. Due to the large number of the overweight, it is expected in the future prevalence of obesity will see more substantial growth. Obesity can increase the risk of incidence by more than 3-fold in type 2 diabetes, cholecystitis, hyperlipidemia and insulin resistance by more than 3 times, and by 2 to 3 times in coronary heart disease, hyperuricemia, gout, osteoarthritis .In addition, overweight and obesity can cause a series of social and psychological problems.

Studies have shown that moderate weight loss in obese individuals (5% -10%) can reduce the risk of obesity-related disease [2]. In the existing means of weight intervention, a reasonable diet and exercise is an effective method of weight control. Eight RCT-based meta-analyzes showed that improving dietary patterns resulted in appropriate, clinically significant weight loss. Four meta-analyzes based on RCTs also showed that physical activity intervention resulted in changes in exercise habits and improved cardiorespiratory function [3]. However, as the incidence of obesity gradually increased, the obesity population expands rapidly; Lifestyle intervention, although effective, is poorly adhered, has small coverage for individual or small-unit groups, and is often time-consuming, laborious with low cost-effectiveness and little

control over the entire obesity population. In recent years, IT network communication technology development and popularization provides a new way of thinking for weight management and weight loss method. IT technology is widely used, and has the characteristics of almost ubiquitous penetration. Therefore it can achieve real-time communication with the large crowd, saving a lot of human resources and costs while achieving long-term follow- supervision and dynamic management. In the early stage, our lab was involved in a project with Duke University, USA, which used a daily program sending text messages to the subjects and the ordinary pedometer as a monitoring tool in Beijing for 6 months of clinical randomization Controlled study. The results show that the use of SMS-assisted life-style intervention have a significant effect on reducing overweight body weight, waist circumference, blood pressure and other indicators [4]. But the general pedometer can only record the total number of daily steps but not the appropriate strength and effective amount of exercise. Therefore, study on how to objectively monitor the use of scientific and effective exercise is warranted.

The incidence of obesity is affected by a variety of factors, including genes. In addition to individual differences in compliance, there are differences in individual gene levels. Therefore, lifestyle interventions are not effective for everyone. The PLIN gene is one of the candidate genes for obesity, and the encoded adipocyte-associated protein, Perilipin, is a phosphoprotein that can be coated on the surface of fat droplets in adipocytes. The PLIN gene in human is located at 15q26.1 and is susceptible to obesity, diabetes and hypertriglyceridemia [5]. The studies showed that PLIN SNPs were related to the risk of obesity and glycometabolism and lipid metabolism, and the effects of diet intervention and exercise intervention on weight loss of obese people were different. The effects of different genotypes on weight loss after intervention were different. At present, there are 218 polymorphisms of human PLIN gene at home and abroad, of which PLIN1 (6209T> C), PLIN3 (17071A> T), PLIN4 (11482G> A), PLIN5 (13041A> G), PLIN6 (14995A> T) and PLIN7 (13042A> G). The distributions of PLIN gene single

nucleotide polymorphisms (SNPs) in the obese population and the relationship with the effect of weight loss was preliminarily studied in our lab. The results showed that PLIN1 was the most common type of obesity in Chinese Han adults. C phenotype might be associated with obesity risk. PLIN4 was mainly expressed in common G phenotype. The phenotype of rare gene A might be associated with low BMI and low Obesity risk; PLIN6 common A gene phenotype common, rare gene T phenotype may be related to adult women with low BMI and low risk of obesity [6]. Therefore, it is of importance to explore the distribution of PLIN SNP in the population and its interaction with lifestyle, and to carry out individualized treatment of obesity.

The purpose of this study is to establish a lifestyle intervention for obesity based on IT management platform and prescription pedometer application, to observe the role of these technologies in the prevention and treatment of obesity, to explore the weight management and weight loss of the best effective exercise and individual exercise prescription, and for the real-time weight management and obesity intervention and individualized treatment technology to provide a scientific basis.

2 Objective

Main Objective: To explore the IT technology management platform-based and prescription pedometer-assisted exercise on weight management for Chinese adults, and to find the minimum effective amount of exercise on weight loss for obese adults.

Secondary Objective: To observe the effects of lifestyle intervention and obesity gene (PLIN gene) interaction on weight management in Chinese adults.

3 Study Design

3.1 Subjects

The subjects of this study are18-65-year-old Chinese obesity and overweight adults and people who are willing to manage their weight or improve their lifestyle. Refer to "Chinese adults overweight and obese BMI Screening Criteria", We used BMI to assess underweight (<18.5 kg/m^2), normal-weight (⩾18.5 to 23.9 kg/m^2), overweight (⩾24.0 to 27.9 kg/m^2), and obesity (⩾28 kg/m^2). Exclusion criteria:1) Secondary obesity, and 2) history of weight-loss interventions (bariatric surgery, acupuncture, moxibustion, or prescribed medication, etc.) and significant changes in body weight in the past three months; 3) diagnosed osteoarthritis , abnormal physical deformities and history of spine or limbs surgery or fracture in the past three months; 4) severe functional disorder or organic diseases of the heart, liver or kidney; 5) uncontrolled hypertension (blood pressure more than 180 / 110mmHg) or its complications; 6) fasting blood glucose more than 16.7mmol / L or diabetic complications; 7) pregnant or planned within 10 months of pregnancy, lactating women.

3.2 Study design

This study is self-controlled open intervention study. We intended to choose the occupational groups and business enterprises as a unit, and to take cluster sampling and advertising for recruitment of volunteers.

3.3 Research procedures

1. To establish the IT technical platform of lifestyle intervention

(1) Intervention program

First of all, the basic information of the subjects was collected and evaluated according to the information provided and classified according to gender, age, BMI and so on. Then, according to the current physical activity level and cardiopulmonary function of different groups, the two stages of exercise prescription and diet prescriptions were developed respectively, and each stage is divided into three levels; and regular evaluation of the completion of the prescription was performed.

(2) IT platform

We cooperate with Descom Information Technology (Beijing) Co., Ltd., to develop a health management system application software to incorporate the intervention program and finally establish the lifestyle intervention health management IT technology platform.

2. Using the IT technology platform to conduct the weight management and obesity intervention research

Based on the results of the medical evaluation, personalized exercise prescriptions and nutritional guidance were generated, and the exercise prescriptions were downloaded to the prescribing pedometer. Intervention period was 3 to 6 months.

Intervention

(1)Exercise intervention: Aerobic exercise based and supplemented by resistance training and appropriate flexibility exercise. The intensity or duration of exercise increased month by month gradually at first three months.

Exercise intensity: moderate intensity, in steps / minute;

Exercise type: the brisk walking, supplemented by muscle strength training (resistance movement) and flexibility training;

Exercise duration: 30-90min per day, the specific time to exercise personalized prescription shall prevail.

Exercise frequency: more than 3 days per week

According to the initial physical activity level, the individual exercise prescription is formulated and the exercise prescription is downloaded to the pedometer to remind the subject to exercise according to the strength and time required by the prescription. If the prescription rate is better (⩾ 80%), then the intensity of exercise and exercise in the first 3 months by the gradient increasing month by month.

(2)Dietary prescription: Limited energy balance diet

Controlled energy balance diet: according to the standard weight and physical activity level to determine the total energy intake, given dietary prescriptions and recipe examples. Three major nutrients for energy than: fat of 20-25% of fat, carbohydrates of 50-60% and protein of 15-20%.

(3) Health education: Health education was conducted before and after the intervention, including balanced diet, reasonable nutrition, healthy eat, scientific exercise, safe exercise, etc.

(4)Regular and real-time monitoring: The use of pedometer for real-time monitoring of exercise, and using the questionnaire on resistance movement and dietary regular to remind and adjust the diet structure and behavior 1time / 3month.

3.4 Follow-up Plan

Study Period	Screening	included	M1		M2		M3		M4		M5		M6	
Study week	week -8—-4	week -4—0	wk 1-3	wk4	wk 5-7	wk 8	wk 9-11	wk 12	wk 13-15	wk 16	wk 17-19	wk 20	wk 21-23	wk 24
Inform consent	√													
Inclusion &exclusion	√													
Basic information		√												

Physical examination		√						√						√
Lifestyle Questionnaire		√						√						√
Dietary survey		√						√						√
IPAQ(long last)		√						√						√
IPAQ(short last)		√						√						√
Weekly exercise monitoring			√	√	√	√	√	√	√	√	√	√	√	√
Health education		√						√						
PLIN SNPs		√												
BP, FBG		√						√						√
Blood Serum chemistry		√						√						√
Adverse event monitoring		√	√	√	√	√	√	√	√	√	√	√	√	√
Drug combination		√	√	√	√	√	√	√	√	√	√	√	√	√

3.5 Outcome and its measurement

Measurements included baseline, intervention for 3 months (medium term), and interventions for 6 months:

Main indicators:

Physical examination: height, weight, body fat (body composition analyzer), waist circumference (refer to WHO waist measurement method); BMI, waist / height.

Secondary indicators:

Lifestyle, diet and physical activity level: lifestyle questionnaire, food frequency questionnaire,

IPAQ questionnaire;

Blood pressure: mercury vertical sphygmomanometer, upper arm electronic sphygmomanometer (Omron);

Fasting blood glucose, OGTT and blood lipids: automatic blood biochemical analyzer;

Serum Insulin: ELISA;

Plasma CRP, adiponectin, leptin: ELISA

PLIN1, PLIN4, PLIN6 locus single nucleotide polymorphisms: one generation sequencing method.

3.6 Sample size calculation

The sample size was calculated according to the formula of $N=2\times[(Z\alpha+Z\beta)\times\delta/d]^2$, the preliminary study of the SNP distribution of obese adults in Han nationality, and drop-out rate (20%). About 348 subjects are required for each group, assuming a two-sided test of $\alpha = 0.05$ and β of 80%, which indicates that 700 subjects in total should be included in this trial to ensure the quality of the test.

4 Data Management

4.1 Data input

The basic information of the subjects and corresponding questionnaires were entered and stored through the IT platform at baseline. Other biochemical and genotypic information will be entered by the test performer in a timely and accurate manner after completion of the test. The amount of exercise data from the subjects will be pedometer data uploaded to the network through the USB interface, research professionals through the IT technology management platform to upload data on a regular basis to collect and evaluate.

4.2 Data verification and management methods

After the researchers completed the information collection and recording, the data will be submitted to the database administrator through the IT platform for review, integration, and the data administrator will write an inspection report, which includes the completion of the subject (including the loss of information), selected / excluded Standard check, integrity check, and adverse event check and so on.

4.3 Data archiving

Upon completion of the data entry and verification of the questionnaire, the questionnaires are kept in the order of the subject number for examination.

Electronic data files, including databases, analysis programs, analysis results, etc. should be categorized to save and have multiple backups stored on different disks or recording media to prevent damage.

5 Statistical Analysis

The distribution of genes was analyzed by Hardy-Weinberg equilibrium test. Gene linkage was analyzed by Haploview software and Linkage-Disequilibrium analysis was used. Depending on demand, the statistical analysis methods including t-test, ANOVA and non-parametric tests were used for each result of the physical and blood biochemical indicators; and using baseline data carried forward imputation method for handling missing data for ITT analysis. All statistical analyzes were performed using a bilateral test, $P < 0.05$ was considered to test the difference was statistically significant.

6 Safety Assessment

6.1 Adverse events (AE) and serious adverse events (SAE)

Adverse events that may occur in this study are mainly due to sports injury caused by improper exercise, but anything that may occur during the course of the study that results in a failure to participate in the trial should be carefully recorded and reported promptly.

6.2 Reporting of serious adverse events

In the event of any adverse event, whether or not related to the study intervention, and whether intervention has been implemented, the investigator must be notified by phone or SMS within 24 hours of the serious adverse events. This study is open, remote monitoring of the management, if the subjects did not report adverse events to the researchers; the default is no adverse events.

7 Subjects Protection

The protocol and informed consent have been approved by the Biomedical Ethics Committee of Peking University Third Hospital. During recruitment period, the researcher has fully explained and explained the research background, nature, significance, steps, benefits, risks, compensation, and withdrawal and so on. After then, the subjects voluntarily agree and sign the informed consent form and carry out the research. Any adverse events that occurred during the study period were reported promptly and required to be properly addressed.

8.1 Protocol Amendment

If the inspector detected persistence of non-adherence to the inclusion criteria, or if the recruitment criteria were too stringent to meet the needs of the sample size, the study protocol will be amended in this section. Because the corresponding revisions may affect the results, it may also requires a sample size adjustment or an amendment to the analysis plan.

8.2 Premature trial termination

The definition of the principle of early termination of the study and the related measures, following:

1. Serious safety problems were identified in the trial and clinical trials should be discontinued promptly.
2. Significant errors in the clinical trial protocol were found during the trial or serious discrepancies were found in the practice, making it difficult to assess the intervention effect.
3. The applicant of the study or administration department requested termination of the trail.

9 Processes and Progress

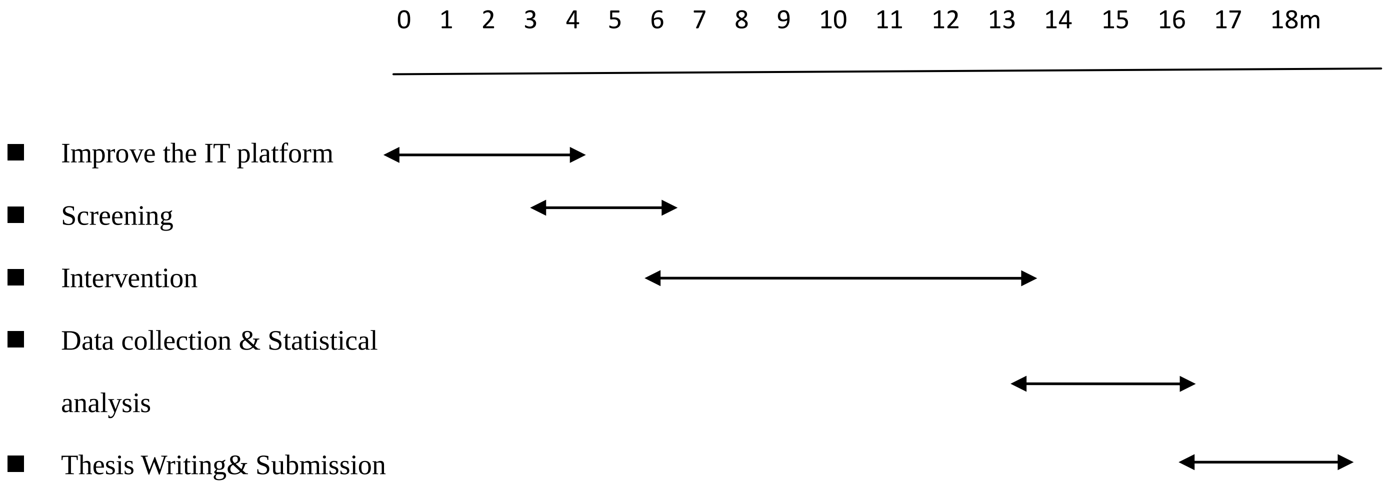

10 References

[1] Lin X, Li HX, Ye XW et al. Study of environmental and genetic factors with metabolic diseases in Chinese population. Chinese Bulletin of Life Sciences, 2012, 24(07): 614-625.

[2] National Heart Lung and Blood Institute (NHLBI). Clinical Guidelines on the identification, evaluation, and treatment of overweight and obesity in adults–the evidence report (ClinicalGdlns). Obes Res 1998; 6: 51S–209S.

[3] Curioni CC, Lourenco PM, Long-term weight loss after diet and exercise: a systematic review. Int J Obes (Lond), 2005, 29(10): 1168-74.

[4] Lin PH, Wang Y, Levine E, et al. A text messaging-assisted randomized lifestyle weight loss clinical trial among overweight adults in Beijing. Obesity (Silver Spring), 2014, 22(5): E29-37.

[5] Rankinen T, Zuberi A, Chaqnon YC, et al. The human obesity gene map: the 2005 update. Obesity (Silver Spring), 2006, 14(4): 529-644.

[6] Chen YB, Chang CQ, Huang ZZ, et al. Distribution of PLIN Gene Polymorphism in Chinese Han Obese Adults. Acta Nutrimenta Sinica, 2011,33(01): 29-33.
